# Mitochondrial Proteome of Affected Glutamatergic Neurons in a Mouse Model of Leigh Syndrome

**DOI:** 10.3389/fcell.2020.00660

**Published:** 2020-07-28

**Authors:** Alejandro Gella, Patricia Prada-Dacasa, Montserrat Carrascal, Andrea Urpi, Melania González-Torres, Joaquin Abian, Elisenda Sanz, Albert Quintana

**Affiliations:** ^1^Mitochondrial Neuropathology Lab, Institut de Neurociències, Universitat Autònoma de Barcelona, Bellaterra, Spain; ^2^Department of Biochemistry and Molecular Biology, Universitat Autònoma de Barcelona, Bellaterra, Spain; ^3^Department of Cellular Biology, Physiology and Immunology, Universitat Autònoma de Barcelona, Bellaterra, Spain; ^4^Proteomics Laboratory CSIC/UAB, Institute of Biomedical Research of Barcelona, Spanish National Research Council (IIBB-CSIC/IDIBAPS), Barcelona, Spain

**Keywords:** Leigh syndrome, animal models, neuroscience, proteomics, cell type-specific, mitochondrial isolation

## Abstract

Defects in mitochondrial function lead to severe neuromuscular orphan pathologies known as mitochondrial disease. Among them, Leigh Syndrome is the most common pediatric presentation, characterized by symmetrical brain lesions, hypotonia, motor and respiratory deficits, and premature death. Mitochondrial diseases are characterized by a marked anatomical and cellular specificity. However, the molecular determinants for this susceptibility are currently unknown, hindering the efforts to find an effective treatment. Due to the complex crosstalk between mitochondria and their supporting cell, strategies to assess the underlying alterations in affected cell types in the context of mitochondrial dysfunction are critical. Here, we developed a novel virus-based tool, the AAV-mitoTag viral vector, to isolate mitochondria from genetically defined cell types. Expression of the AAV-mitoTag in the glutamatergic vestibular neurons of a mouse model of Leigh Syndrome lacking the complex I subunit *Ndufs4* allowed us to assess the proteome and acetylome of a subset of susceptible neurons in a well characterized model recapitulating the human disease. Our results show a marked reduction of complex I N-module subunit abundance and an increase in the levels of the assembly factor NDUFA2. Transiently associated non-mitochondrial proteins such as PKCδ, and the complement subcomponent C1Q were also increased in *Ndufs4*-deficient mitochondria. Furthermore, lack of *Ndufs4* induced ATP synthase complex and pyruvate dehydrogenase (PDH) subunit hyperacetylation, leading to decreased PDH activity. We provide novel insight on the pathways involved in mitochondrial disease, which could underlie potential therapeutic approaches for these pathologies.

## Introduction

Severe alterations of the mitochondrial machinery involved in energy generation lead to a group of progressive and usually fatal pathologies collectively known as primary mitochondrial disease (MD), affecting 1:4,000 births (Gorman et al., 2015). Currently, there is no cure and the treatments available are mostly ineffective (Schon et al., 2010). MD predominantly affects high energy-requiring organs such as the brain (Vafai and Mootha, 2012). Indeed, neurological damage plays a prominent role in MD pathology and lethality (DiMauro and Schon, 2008). However, not all neuronal populations are equally vulnerable to MD, but rather show a striking anatomical and cellular specificity (Dubinsky, 2009). Recent reports have evidenced the remarkable regional heterogeneity in mitochondrial protein composition in the brain, which may account for the cell type specificity of MD (Pagliarini et al., 2008).

Humans harboring mutations in NDUFS4, a subunit of the mitochondrial complex I involved in the assembly and stability of the complex (Scacco et al., 2003; Kruse et al., 2008; Calvaruso et al., 2012) develop Leigh Syndrome (LS), a severe form of MD (DiMauro and Schon, 2008). These individuals die at an early age, commonly presenting brain lesions, hypotonia and respiratory deficits (Leshinsky-Silver et al., 2009; Lombardo et al., 2014). Mice lacking this protein (NDUFS4KO) recapitulate the human symptoms and are an excellent correlate of the human pathology (Quintana et al., 2010; Breuer et al., 2013). As observed in LS patients, NDUFS4 deficiency in mice leads to prominent bilateral lesions in the dorsal brainstem, more specifically in the vestibular nucleus (VN). Conditional NDUFS4 ablation restricted to the VN causes a pathology similar to global Ndufs4KO mice. Conversely, viral-mediated post-weaning re-expression of *Ndufs4* in the VN is sufficient to ameliorate the pathology and to extend the median lifespan of Ndufs4KO mice by 30% (Quintana et al., 2012a). In addition, NDUFS4 deficiency reveals a cell type-specific susceptibility to the disease. NDUFS4 ablation in glutamatergic neurons (Vglut2-expressing) recapitulates most of the phenotype present in the global NDUFS4-deficient mice (Bolea et al., 2019), thus warranting further study of glutamatergic neurons in the VN.

Here, we have developed and implemented a novel viral vector approach (AAV-mitoTag) to purify cell type-specific mitochondria in a fast and minimally disruptive manner. Other genetic tools to isolate mitochondria with cell-type specificity have recently been developed (Bayraktar et al., 2019; Fecher et al., 2019). However, these rely on breeding strategies with mouse lines that have the potential disadvantage of introducing experimental confounds, such as germline recombination or transient expression of Cre recombinase during development, therefore compromising the cell type specificity of these approaches (Lam et al., 2011; Sanz et al., 2015). Cre-dependent viral vector approaches overcome these limitations since only adult cells expressing Cre recombinase are targeted.

The implementation of this approach in glutamatergic neurons in the VN has allowed us to gain a better understanding of both mitochondrial proteome and acetylome specifically in a subset of cells highly susceptible to mitochondrial dysfunction. Hence, we provide insight on the specific proteomic changes driven by NDUFS4 deficiency, which may underlie the molecular and metabolic alterations elicited by mitochondrial dysfunction, with the overarching goal of identifying novel molecular targets with therapeutic potential for LS and MD.

## Materials and Methods

### Animal Husbandry

Mice were maintained with Teklad Global rodent diet No. 2018S (HSD Teklad Inc., Madison, Wis.) and water available *ad libitum* in a vivarium with a 12-h light/dark cycle at 22°C. All experiments were conducted following the recommendations in the Guide for the Care and Use of Laboratory Animals and were approved by the Animal Care and Use Committee of the Universitat Autònoma de Barcelona (Barcelona, Spain).

### Mouse Genetics

*Slc17a6*^Cre^ (BAC-Vglut2::Cre) mice were generated by Ole Kiehn (Borgius et al., 2010). *Ndufs4*^lox/lox^ and *Ndufs4*^Δ/+^ were previously generated by our group (Kruse et al., 2008; Quintana et al., 2010). Mice with conditional deletion of *Ndufs4* in Vglut2-expressing glutamatergic neurons (*Slc17a6*^Cre^, *Ndufs4*^Δ^^/lox^ or Vglut2:Ndufs4cKO) were generated by crossing mice with one *Ndufs4* allele deleted and expressing a codon-improved Cre recombinase (iCre) under the *Slc17a6* promoter (*Slc17a*6^Cre^, *Ndufs4*^Δ/+^) to mice with two floxed *Ndufs4* alleles (*Ndufs4*^lox/lox^). Littermate controls were *Slc17a6*^Cre^, *Ndufs4*^lox/+^ (Vglut2:Ndufs4cCT).

### Cell Cultures

HEK293T cells were obtained from the American Type Culture Collection (ATCC). Cells were grown in high glucose Dulbecco’s Modified Eagle Medium (Gibco) supplemented with 10% (v/v) fetal calf serum (Gibco), L-glutamine (Gibco) and penicillin/streptomycin (Gibco) and maintained at 37 °C with 5% CO_2_. Cells were seeded at 200,000 cells/mL and transfected with plasmids with calcium phosphate and/or transduced with adeno-associated viral vectors (AAVs) for 4 days prior to immunostaining for hemagglutinin (HA), western blot analysis or mitochondria immunoisolation (mitoTag) assays.

### Immunofluorescence

For immunofluorescence analysis of cultured cells, HEK293T cells were fixed in 4% paraformaldehyde (PFA) for 10 min at RT, and further washed two times with PBS before permeabilization with 0.25% Triton X-100 in PBS for 10 min. After permeabilization, cells were washed three times for 5 min with PBS, blocked with 1% BSA in PBS-Tween 20 (0.05%) for 60 min, and incubated with an anti-HA antibody (at 1:1,000 dilution, #901514 BioLegend) overnight at 4°C. After incubation, cells were washed three times with PBS for 5 min and further incubated with anti-mouse Alexa 555 antibody (1:500, Thermo Fisher Scientific). Finally, cells were washed three times with PBS for 5 min, counterstained with Hoechst 33258 (2 μg/mL, Sigma-Aldrich) in PBS for 10 min and mounted onto slides using Fluoromount G (Electron Microscopy Sciences) before microscopic analysis. Immunofluorescence analysis was accomplished in mouse brains fixed overnight in 4% PFA in PBS (pH 7.4). Subsequently, brains were cryoprotected in a PBS solution containing 30% (w/v) sucrose and frozen in dry ice. Frozen brains were embedded in OCT, sectioned at 30 μm in a cryostat and rinsed in PBS-Tx buffer (Phosphate-buffered saline containing 0.2% (v/v) Triton X-100). Non-specific binding was blocked with 10% (v/v) normal donkey serum (NDS) in a PBS-Tx solution for 60 min at room temperature, followed by overnight incubation at 4°C with primary antibodies diluted in 1% NDS-PBS-Tx (1:1,000 for mouse anti-HA, #901514 BioLegend; 1:1,000 for rabbit anti-TOM20, #sc-11415 Santa Cruz Biotech; 1:1,000 for rabbit anti-VDAC, #ab154856 Abcam; 1,000 for Alexa Fluor 488 anti-HA, #901509 Biolegend). Sections were then washed in PBS-Tx and incubated for 1 h at room temperature with the corresponding Alexa Fluor-conjugated secondary antibodies (1:500, Thermo Fisher Scientific) in 1% NDS-PBS-Tx. Sections were finally washed in PBS-Tx and rinsed in water before mounting onto slides with Fluoromount G with DAPI (Electron Microscopy Sciences) for microscopic analysis.

### Western Blotting

For western blot analysis of cultured cells, HEK293T cells were lysed with SDS sample buffer (62.5 mM Tris–HCl, pH 6.8, 2% SDS, 10% glycerol, all from Sigma-Aldrich) without dithiothreitol (DTT) and bromophenol blue, to avoid interference with protein quantification. Prior to protein determination, samples were sonicated to shear genomic DNA and reduce the viscosity of the lysates. Protein concentration of the lysates was determined using the BCA protein assay (Thermo Fisher Sci.) according to the manufacturer’s instructions. After protein quantification, 50 mM DTT and 0.1% bromophenol blue (Sigma-Aldrich) were added to the samples. Lysates from mitoTag assays were obtained as described below. Western blot analysis of whole tissue lysates or immunoprecipitates from mitoTag assays was accomplished by adding 4X Laemmli sample buffer to samples prior to heat-denaturing. Protein lysates (10 μg for HEK293T total cell lysates, 0.125% whole tissue lysates and 10% immunoprecipitates for mitoTag assays in HEK293T cells, and 2 μg for whole tissue lysates and immunoprecipitates for mitoTag assays in tissue were heated at 99°C for 3 minutes and subjected to 4–20% gradient SDS-PAGE prior to transfer to nitrocellulose membranes (Bio-Rad Laboratories, Inc.). Membranes were then blocked for 1 h with 5% (w/v) dried skimmed milk in TBS-T buffer (Tris-buffered saline containing 0.1% (v/v) Tween-20) and incubated overnight at 4°C with primary antibodies against HA (1:1,000; #715500 Invitrogen), TOM20 (1:5,000; #sc11415 Santa Cruz Biotech), NDUFS4 (1:500: #87399 Abcam), ATPIF1 (1:1,000; #A-21355 Invitrogen), GAPDH (1:1,000; #GTX627408 GeneTex), Calreticulin (1:1,000; #ab2707 Abcam) and PDHA1 (1:1,000; ab155096 Abcam). After incubation with the corresponding HRP-conjugated secondary antibodies (1:10,000; Jackson ImmunoResearch), membranes were washed in TBS-T and developed using an enhanced chemiluminescence (ECL) detection system (Thermo Fisher Sci.) in a C-DiGit Blot Scanner with Image Studio Ver 5.2 (Licor). Bands were quantified using Image Studio (Licor).

### Double-Labeled *in situ* Hybridization (ISH)/ Immunofluorescence (IF)

For double-labeling *in situ* hybridization (ISH)/immunofluorescence (IF) assays, fresh brainstem sections containing the VN were embedded in OCT, frozen in dry ice and stored at −80°C until sectioning. Coronal sections (16 μm) were obtained using a cryostat and used for analysis. Double-labeled ISH/IF was accomplished by performing an RNAscope assay for *Slc17a6* (Vglut2) transcripts followed by immunofluorescence analysis for HA. RNAscope assay for *Slc17a6* (#319171 Advanced Cell Diagnostics) was performed according to manufacturer’s directions. After *Slc17a6* detection, sections were incubated overnight at 4°C with an anti-HA antibody (1:1,000; #715500 Invitrogen) followed by a 1 h incubation at room temperature with an anti-rabbit Alexa Fluor 488-conjugated secondary antibody (1:500, Thermo Fisher Scientific). Sections were finally washed in PBS-Tx and rinsed in water before mounting onto slides with Prolong Diamond with DAPI (Thermo Fisher Scientific) for microscopic analysis.

### Transmission Electron Microscopy

Vestibular nuclei mitochondria were fixed in 2.5% (v/v) glutaraldehyde (Merck) in 0.1 M phosphate buffer (PB), pH 7.4 at 4°C overnight. Postfixed for 2 h with 1% (w/v) osmium tetroxide (TAAB Lab) containing 0.8% (w/v) potassium hexacyanoferrate (Sigma-Aldrich) in PB, dehydrated in gradual ethanol (50–100%), embedded in Eponate 12 resin (Ted Pella Inc.), and polymerized at 60°C for 48 h. Ultrathin sections of 50 nm were collected onto carbon-coated copper grids of 200 mesh and contrasted with conventional uranyl acetate and lead citrate solutions. Sections were visualized in a TEM Jeol JEM-1400 (Jeol Ltd., Tokyo, Japan) operating at 80 kV and equipped with a CCD Gatan ES1000W Erlang Shen camera (Gatan Inc.).

### MitoTag Adeno-Associated Viral Vector (AAV) Generation

We generated an adeno-associated virus (AAV) that would initiate Cre-inducible mitoTag expression using a double floxed inverted open reading frame (DIO) approach. An NheI/AscI fragment containing a construct coding for the mouse *Tomm20* gene fused to three hemagglutinin (HA) sequences (Tomm20 ⋅3HA) was cloned into a NheI/AscI-digested pAAV-EF1a-DIO-WPRE-hGH polyA plasmid to obtain a pAAV1-EF1a-DIO-Tomm20⋅3HA- WPRE-hGH polyA construct (AAV-mitoTag). An AAV (AAV1 serotype) vector was produced and CsCl-gradient purified as described previously (Quintana et al., 2012a). The TOM20⋅HA fusion protein was generated by mutation of *Tomm20* stop codon to glutamic acid (E) and phenylalanine (F).

### AAV-mitoTag Delivery

Mice (*n* = 28, 37.4 ± 7 days, 22.1 ± 3.2 g) were anesthetized with isoflurane (5.0 % induction, 1.5–0.7 % maintenance) and placed in a stereotaxic frame (Kopf Instruments). Ketoprofen analgesic solution (5 mg/kg s.c; Sanofi-aventis) and ocular protective gel (Viscotears^®^) were applied before stereotaxic procedure. Mice received intracranial AAV injections into the vestibular nucleus (VN) at the following coordinates: AP −0.6, ML ± 0.125, DV −0.39 mm from the Bregma, using the correction factor (Bregma - Lambda/4.21). A total of 1.0 μL of AAV-mitoTag vector (0.7 × 10^12^ viral genomes/mL; 0.5 μL per side) was injected into each hemisphere at a rate of 0.25 μL/min using a 5 μL Hamilton syringe with a 32G blunt needle. Following injection, the needle was kept in place for 4 min post-delivery to allow proper viral vector diffusion and, subsequently, removed at 1 mm/min.

### Isolation of Mitochondria by Immunoaffinity Purification

For HEK293T cells, 200,000 cells/mL were transduced with AAV-mitoTag and a viral vector expressing Cre recombinase (AAV-Pkg-Cre; UNC Vector Core) or left untransduced for 4 days prior to lysis in mitochondria isolation kit lysis buffer (1 × 10^7^ cells/mL; Miltenyi Biotec) with protease inhibitors (Sigma-Aldrich) and homogenized by passing the lysate 25 times through a 1 mL syringe with a 25G needle. Homogenized lysates were next centrifuged at 700 x g for 10 min at 4°C, and supernatants collected and incubated with anti-HA or anti-TOM22 microbeads (Miltenyi Biotec) according to manufacturer’s directions. Mitochondria from glutamatergic cells in the vestibular nucleus (VN) were isolated from freshly collected mouse brain tissue. Tissue homogenates were prepared using the Mitochondria Extraction Kit, tissue (Miltenyi Biotec) according to manufacturer’s instructions. Briefly, following decapitation, brains were weighted, and vestibular nuclei dissected according to the Paxinos mouse brain atlas and digested with extraction buffer for 30 min at 4°C. After centrifugation at 300 × *g* for 5 min at 4°C, pellets were resuspended in Solution 2 supplemented with EDTA-free protease inhibitor cocktail (Roche Diagnostics), and, for acetylome analysis assays, deacetylase inhibitors (10 mM nicotinamide, 10 μM TSA and 10 mM sodium butyrate). Digested tissue was homogenized using a glass dounce homogenizer. Homogenates were centrifuged at 500 × *g* for 5 min at 4°C. Subsequently, supernatants containing mitochondria were further purified as directed in the Mitochondria Isolation Kit (Miltenyi Biotec). For magnetic labeling and isolation, supernatants diluted in Separation Buffer were incubated with 50 μL of anti-HA Microbeads on a shaker (60 rpm) for 1 h at 4 °C. Subsequently, LS columns (Miltenyi Biotec) were placed in a magnetic QuadroMACS^TM^ Separator (Miltenyi Biotec) and equilibrated with 3 mL Separation Buffer (Miltenyi Biotec). Microbead-coated mitochondria were applied onto the LS column, followed by three 3-mL washing steps with Separation Buffer. Columns were removed from the separator and mitochondria gently flushed out in 1.5 mL of Separation Buffer with a plunger. Mitochondria were pelleted by centrifugation for 2 min at 12,000 × *g* and washed twice with Storage Buffer. Two immunoprecipitation (from 4–5 mice) were pooled for proteomic analysis.

### Isolation of Mitochondria by Double Centrifugation

Mitochondria were isolated by differential centrifugation using protocols previously described (Brown et al., 2004; Pagliarini et al., 2008). Briefly, following decapitation, vestibular nuclei were dissected according to the Paxinos mouse brain atlas and homogenized using a glass dounce homogenizer containing MAS buffer (70 mM sucrose, 220 mM mannitol, 10 mM KH_2_PO_4_, 5 mM MgCl_2_, 1 mM EGTA, 2 mM HEPES-KOH, pH 7.2). Homogenates were centrifuged at 900 × *g* for 10 min at 4°C. Supernatants were further centrifuged at 9,000 × g for 10 min at 4°C. Resulting supernatants were used for L-lactate measurements (Biosystems S.A.), and pellets containing mitochondria were resuspended in MAS buffer. Protein concentration was determined using the BCA protein assay (Thermo Fisher Sci.) according to the manufacturer’s instructions.

### Pyruvate Dehydrogenase (PDH) Activity

PDH activity was measured in VN homogenates by using the Pyruvate Dehydrogenase (PDH) Enzyme Activity Microplate Assay Kit (Abcam) according to manufacturer’s instructions. Briefly, mitochondria isolated by double centrifugation (50 μg) were loaded into the wells of a 96-well plate that was specific for PDH activity assay. PDH enzyme was immunocaptured by the monoclonal antibodies coated on the wall of each well. The enzymatic activity of PDH was determined based on the production of NADH coupled to a reporter dye whose formation is monitored by measuring the increase in absorbance at 450 nm on a Varioskan LUX plate reader (Thermo Fisher Sci.).

### Measurement of Respiration in Isolated Mitochondria

Oxygen consumption rate (OCR) was measured in MAS buffer supplemented with 0.5 % (w/v) fatty acid–free BSA, using a Seahorse XFp Extracellular Flux Analyzer (Agilent Technologies, Inc). Freshly isolated mitochondria (5–20 μg of protein) obtained by double centrifugation were plated in each well of an XFp plate in 25 μL of MAS-BSA Buffer. To attach mitochondria, the plate was centrifuged at 2,000 × *g* at 4°C for 20 min and then 155 μL of MAS-BSA buffer containing 10 mM pyruvate/5 mM malate, 100 μM palmitoyl-L-carnitine/5 mM malate, or 20 mM succinate/4 μM rotenone was added to each well. First, basal measurements of oxygen consumption rate (state II) were obtained. Next, ADP (5 mM) was injected (state III), followed by sequential injections of oligomycin (2 μM, state IV), FCCP (5 μM, state IIIu) and antimycin A/rotenone (2.5 μM and 2 μM, respectively) to disrupt mitochondrial respiration. Together, this injection series allowed for the determination of ADP-uncoupled respiration (ADP), proton leak (oligomycin), maximal respiration (FCCP) and non-mitochondrial residual oxygen consumption (antimycin A/rotenone).

### Protein Extraction and Digestion

Mitochondria from glutamatergic neurons in the vestibular nucleus were immunocaptured as described above. These mitochondria were then suspended in lysis buffer (4% (w/v) SDS, 100 mM Tris–HCl, pH 7.6, 0.1 M DTT) and incubated at 95°C for 5 min. Protein extracts were quantified using RC-DC^TM^ Protein Assay (Bio-Rad). Three biological replicates from Vglut2:Ndufs4cCT and Vglut2:Ndufs4cKO mice mitochondria were processed. Protein was digested with sequencing grade modified trypsin (Promega) using the FASP (Filter Aided Sample Preparation) digestion protocol (Wisniewski et al., 2009). Briefly, 150 μg of protein from each sample were loaded onto a 10-kDa Amicon Ultra-0.5 centrifugal filter (Millipore). The protein mixture was washed three times by adding 200 μL of UA buffer (8 M urea, 0.1 M Tris–HCl pH 8.5) to the filter and centrifuging at 14,000 × *g* for 10 min at 13°C. Next, proteins were alkylated with 100 μL of alkylation buffer (0.05 M IAA, 8 M urea, 0.1 M Tris–HCl pH 8.5) in the dark for 20 min at 25°C. Subsequently, the protein extract was washed three times with 100 μL of UA buffer and three times with 100 μL of 200 mM triethylammonium bicarbonate (TEAB). Trypsin digestion was performed at 37°C for 18 h using an enzyme-to-protein ratio of 5:100. Tryptic peptides were eluted by the addition of 3 × 100 μL of 200 mM TEAB followed by a centrifugation at 14,000 × *g* for 15 min at 13°C.

### Peptide Labeling by Isobaric Tandem Mass Tag

Each tryptic peptide mixture obtained from 150 μg of protein extract was isotopically labeled with the corresponding TMT6plex reagent (Thermo Fisher Sci.) based on the standard procedure. TMT-labeled peptide mixtures were combined in a low-bind 1.5 mL Eppendorf tube, evaporated, and desalted using a C18 SPE cartridge (Agilent Technologies). The SPE eluates were evaporated and resuspended in 50 mM MOPS pH 7.2, 10 mM Na_2_HPO_4_, 50 mM NaCl.

### Acetylated Peptide Enrichment

The TMT-labeled peptide mixtures (900 μg) were enriched for acetylated peptides by immunoaffinity purification using an anti-acetyl lysine antibody following the PTMScan^®^ Pilot Acetyl-Lysine Motif (Ac-K) protocol (Cell Signaling Technologies). Peptides eluted from the enrichment were desalted using PolyLC C18 tips (PolyLC Inc., MD, United States), and resuspended in 5% methanol (1% formic acid) for mass spectrometry analysis.

### LC–MSn Analysis

Peptides were analyzed using an Orbitrap Fusion Lumos Tribrid mass spectrometer (Thermo Fisher Sci.) equipped with a Thermo Scientific Dionex Ultimate 3000 ultrahigh-pressure chromatographic system (Thermo Fisher Sci.) and Advion TriVersa NanoMate (Advion Biosciences Inc.) as the nanospray interface. Peptide mixtures were loaded to a μ-precolumn Acclaim C18 PepMap100 (100 μm × 2 cm, 5 μm, 100 Å, C18 Trap column; Thermo Fisher Sci.) at a flow rate of 15 μL/min and separated using a C18 Acclaim PepMap RSLC analytical column (75 μm × 50 cm, 2 μm, 100 Å, nanoViper). Separations were done at 200 nL/min using a linear ACN gradient from 0 to 40% B in 240 min (solvent A = 0.1% formic acid in water, solvent B = 0.1% formic acid in acetonitrile). The mass spectrometer was set up in the positive ion mode and the analysis was performed in an automatic data dependent mode (a full scan followed of 10 HCD scan for the most abundant signals).

### Database Search and Proteomic Data Analysis

Database search was done using Proteome Discoverer v2.1 (Thermo-Instruments) with a 1% false discovery rate (FDR) and the UniProt 2018-10 database restricted to Mus musculus (mouse) and contaminants. Search parameters were precursor and fragment tolerance, 20ppm; enzyme, trypsin; missed cleavages, 1; fixed modifications, TMTsixplex (N-terminal, K), carbamidomethyl (C); and variable modifications, oxidation (M).

For proteomic data analysis, DanteR (Taverner et al., 2012) was used for relative quantification. Only unique peptides were considered for the analysis. Tandem mass tag (TMT) reporter intensity data were normalized using the total intensity for each label. ANOVA was performed using a linear model and *p*-values were adjusted by using the Benjamini-Hochberg FDR correction. In the acetylome quantification, the ratio of the quantified peptides was normalized using the ratio of the corresponding protein. Differential proteins and peptides were selected using a *p*-value (adjusted) cutoff of 0.1 and a fold change lower than 0.8 (down) or higher than 1.2 (up).

The mass spectrometry proteomic data have been deposited to the ProteomeXchange Consortium^1^ via the PRIDE (Vizcaino et al., 2016) partner repository with the dataset identifier PXD016321.

### Bioinformatics Analysis

Functional enrichment analysis using overrepresentation analysis (ORA) with the Gene Ontology (Biological process) and Pathway (Reactome) databases was accomplished using WebGestalt (Liao et al., 2019)^2^. The STRING (Search Tool for the Retrieval of Interacting Genes/Proteins^3^) algorithm (version 11) was used to build protein–protein interaction networks. Active interaction sources, including text mining, experiments, databases, co-expression, neighborhood, gene fusion and co-occurrence were applied. Only high-confidence interactions (score 0.7) were chosen.

### Statistics

Data are shown as the mean ± SEM. GraphPad Prism v6.0 software was used for statistical analyses. Appropriate tests were selected depending on the experimental design as stated in the figure legends. Statistical significance, when reached, is stated in the figure legend.

## Results

### Generation and Validation of the AAV-mitoTag Viral Vector

**FIGURE 1:**
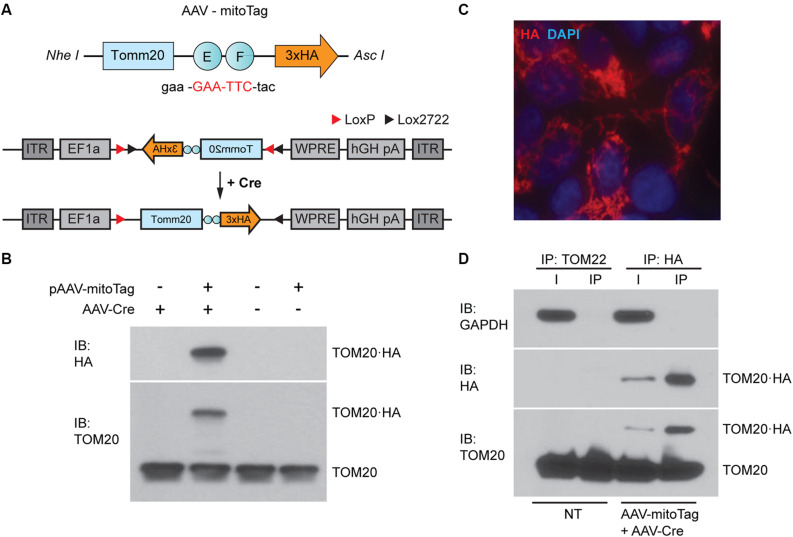
Generation of the mitoTag viral vector (AAV-mitoTag). **(A)** A TOM20⋅hemagglutinin (HA) fusion protein was generated by mutation of *Tomm20* stop codon (red). The resulting construct was cloned in a Cre-dependent viral vector plasmid (pAAV-DIO). **(B)** Western blot analysis showing the expression and Cre-dependency of the pAAV-DIO-TOM20⋅HA construct in HEK293T cells. Immunoblot (IB) for HA and TOM20 shows expression of the fusion protein TOM20⋅HA at the expected molecular weight only in cells transduced with the Cre-expressing viral vector (AAV-Cre). **(C)** Immunofluorescence analysis showing an HA staining consistent with mitochondrial localization of TOM20⋅HA in HEK293T cells. Cells were transfected with the pAAV-DIO-TOM20⋅HA construct and transduced with a viral vector expressing Cre recombinase (AAV-Cre) prior to fixation and immunostaining using an anti-HA antibody. **(D)** Western blot analysis showing specific immunoprecipitation of mitochondria using anti-HA magnetic microbeads. GAPDH was used as a cytosolic marker. TOM20 was used as a mitochondrial marker. Immunoprecipitation assays with anti-TOM22 microbeads were used as a control for mitochondria isolation in non-transduced cells. I: input of the immunoprecipitation; IP: immunoprecipitation; NT: non-transduced.

To allow the isolation of mitochondria from specific neuronal cell types, a Cre-dependent mitoTag adeno-associated viral vector (AAV-mitoTag) was generated by inserting the cDNA of an HA-tagged *Tomm20* into a double-floxed inverse open reading frame (DIO) AAV construct (Figure 1A). Analysis for Cre recombinase-dependency of the construct (Figure 1B) and mitochondrial targeting of the resulting TOM20⋅HA fusion protein (Figure 1C) was first assessed in HEK293T/17 cells. Transfection of the viral vector construct showed that the TOM20⋅HA fusion protein was only expressed in cells transduced with a viral vector expressing Cre recombinase. In these cells, fusion protein showed the expected molecular weight. Immunofluorescence analysis for HA in cells transfected with the mitoTag viral vector construct and transduced with viral vector particles expressing Cre recombinase showed a localization of the fusion protein consistent with incorporation into mitochondria (Figure 1C). To test whether whole mitochondria isolation from Cre-expressing cells could be accomplished by an immunocapture method, lysates from HEK293T/17 cells transduced with the AAV-mitoTag viral vector (AAV-DIO-Tomm20⋅HA) along with a Cre recombinase-expressing viral vector (AAV-Cre) were used to immunoprecipitate HA-tagged mitochondria with anti-HA coated magnetic microbeads (Figure 1D). Anti-TOM22 coated magnetic microbeads were also used as control to immunoprecipitate unlabeled mitochondria from non-transduced cells. Western blot analysis of the immunoprecipitates showed specific immunoisolation of mitochondria without contamination from cytosolic proteins, confirming the suitability of the mitoTag viral vector for the specific purification of mitochondria from Cre recombinase-expressing cells.

### Cell Type-Specific Isolation of Mitochondria From Glutamatergic Neurons in the VN

**FIGURE 2:**
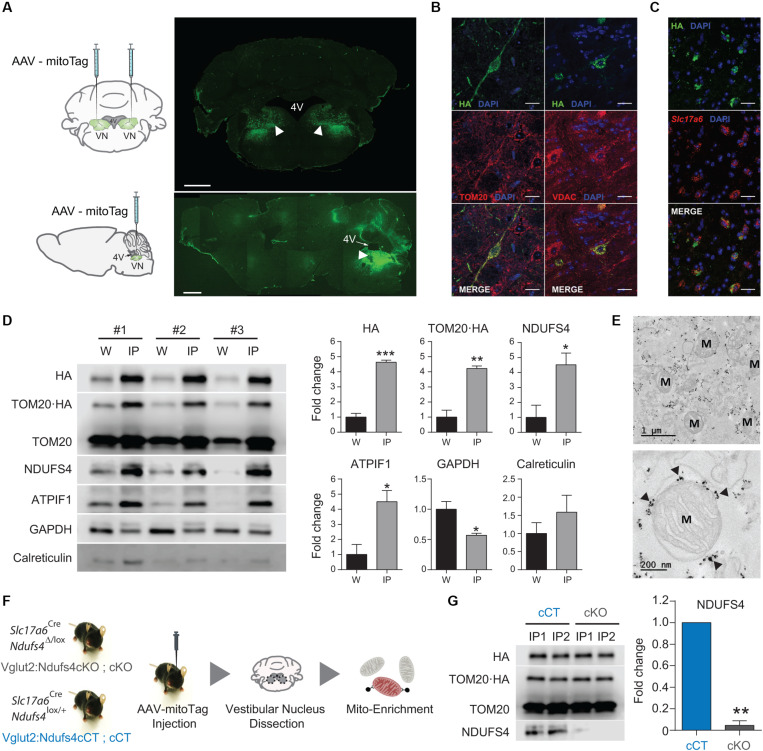
*In vivo* validation of the mitoTag approach. **(A)** Diagram of the targeted injection site (VN; coronal and sagittal view) and immunofluorescence analysis using an anti-HA antibody to confirm transduced neurons in the VN of Vglut2^Cre^ mice. Scale bar: 1 mm. **(B)** Double immunostaining for TOM20/HA and VDAC/HA showing mitochondrial localization of the fusion protein. Scale bar HA/TOM20: 50 μm; Scale bar HA/VDAC: 25 μm; **(C)** Double-label *in situ* hybridization/Immunofluorescence (ISH/IF) assay showing restricted expression of the mitoTag (TOM20⋅HA) in glutamatergic (*Slc17a6*-expressing) cells. Scale bar: 25 μm; **(D)** Western blot analysis and quantification for the relative abundance of the fusion protein TOM20⋅HA and organelle-specific proteins in whole-cell lysates (W) and lysates from mitoTag immunoprecipitates (IP). **(E)** Electron micrographs of mitoTag preparations from the VN of Vglut2:Ndufs4cCT mice. Electron dense spots (black arrows) are anti-HA magnetic microbeads bound to the outer mitochondrial membrane. M, mitochondria. **(F)** Cartoon showing the experimental approach. **(G)** Western blot analysis for HA, TOM20, and NDUFS4 levels and quantification of NDUFS4 in mitoTag immunoprecipitates (IP) from the VN of Vglut2:Ndufs4cCT and Vglut2:Ndufs4cKO mice. Data are presented as the mean ± SEM. Statistical analysis was performed using an unpaired *t*-test (*P < 0.05, **P < 0.01, ***P < 0.001).

Specific deletion of *Ndufs4* in Glutamatergic neurons (Vglut2-expressing) in the VN has been shown to drive motor and breathing deficits in mice (Bolea et al., 2019). To define the underlying mitochondrial alterations induced by complex I deficiency in these cells, we first confirmed the feasibility of the AAV-mitoTag approach for the isolation of mitochondria from this genetically defined neuronal population *in vivo*. To show expression of the mitoTag in mitochondria from the cell type of interest, AAV-mitoTag viral vector was injected bilaterally into the VN of *Slc17a6*^Cre^ (Vglut2^Cre^) mice. Subsequently, brain sections containing the area were analyzed by immunofluorescence using an anti-HA antibody (Figure 2A). HA staining was restricted to specific cells in the VN, and double-labeling analysis using anti-HA and anti-TOM20/VDAC antibodies confirmed the mitochondrial localization of the mitoTag (Figure 2B). Specific expression of the mitoTag in *Slc17a6*-expressing cells was further confirmed by double-label *in situ* hybridization/immunofluorescence (ISH/IF) assays using probes against *Slc17a6* mRNA and an anti-HA antibody (Figure 2C). Immunocapture of mitochondria from Vglut2-expressing cells in Vglut2-Cre mice injected with the mitoTag viral vector was accomplished from lysates of pooled vestibular nuclei using anti-HA coated magnetic microbeads (Figure 2D). Western blot analysis of whole lysates (input of the immunoprecipitation) and immunoprecipitates showed specific enrichment for the fusion protein TOM20-HA (as assessed using anti-HA and anti-TOM20 antibodies) and the mitochondrial markers NDUFS4 and ATPIF1 in the immunoprecipitates. The cytosolic marker GAPDH appeared depleted in the immunoprecipitates. In contrast, the endoplasmatic reticulum (ER) marker calreticulin did not show a significant depletion in this fraction, which is consistent with the fact that mitochondria can form contacts with the ER in neurons (Wu et al., 2017; Figure 2D). Electron microscopy (EM) analysis of the immunoprecipitates from VN lysates of Vglut2^Cre^ mice injected with the mitoTag viral vector confirmed binding of the anti-HA magnetic microbeads to the outer membrane of whole mitochondria (Figure 2E).

### Identification and Quantitation of the Global and Acetyl-Proteome in Mitochondria From Glutamatergic Neurons in the VN

Next, we sought to define the specific protein changes induced by complex I deficiency in mitochondria from glutamatergic neurons in the VN. To accomplish this, mice conditionally deficient for the complex I subunit NDUFS4 in glutamatergic neurons (Vglut2:Ndufs4cKO mice) and their controls (Vglut2:Ndufs4cKO mice) were injected with the AAV-mitoTag viral vector to isolate mitochondria from glutamatergic neurons in the VN (Figure 2F). Western blot analysis of immunoprecipitates revealed specific isolation of HA-tagged, NDUFS4-deficient mitochondria (Figure 2G), which confirms *Ndufs4* deletion in Cre-expressing cells. Cell type-specific analysis of the global mitochondrial proteome and acetylproteome from Vglut2:Ndufs4cKO and Vglut2:Ndufs4cCT mice was accomplished by liquid chromatography-tandem mass spectrometry (LC–MSn). Global proteome and acetylome were simultaneously identified and quantified from pooled mitoTag preparations from the VN of Vglut2:Ndufs4cKO (*n* = 3) and Vglut2:Ndufs4cCT mice (*n* = 3). Each replicate was a pool of two mitoTag preparations. 95% of labeled peptides were dedicated to analysis of the acetyl-proteome, the remaining 5% was used for global proteome analysis.

**FIGURE 3:**
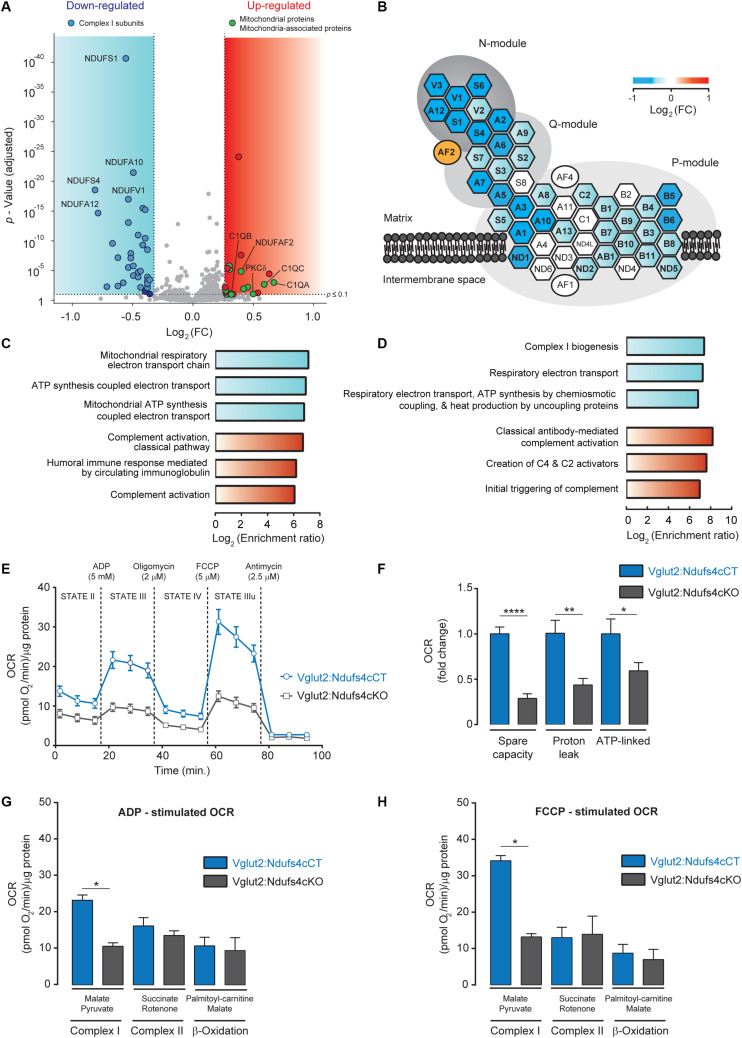
Analysis of the mitochondrial proteome and respiration in mitochondria from the VN of Vglut2:Ndufs4cKO mice. **(A)** Volcano plot showing proteomic changes induced by NDUFS4-deficiency in mitoTag preparations from Vglut2:Ndufs4cKO and control mice. Blue area contains proteins (*n* = 34) with decreased abundance (< 0.8-fold, P adj < 0.1) and red area contains proteins (*n* = 22) with increased abundance (> 1.2-fold, P adj < 0.1). **(B)** Schematic illustration of mitochondrial complex I structure. Heat map shows the changes induced by NDUFS4-deficiency in complex I proteins from panel **(A)**. Hexagons represent complex I subunits and ovals represent complex I assembly factors. For the sake of clarity, prefix “NDUF” has been omitted. **(C)** Enriched biological process GO terms for proteins with decreased (blue) or increased (red) abundance (FDR < 0.05) in mitoTag preparations from the VN of Vglut2:Ndufs4cKO mice. **(D)** Enriched pathways (Reactome) for proteins with decreased (blue) or increased (red) abundance (FDR < 0.05) in mitoTag preparations from the VN of Vglut2:Ndufs4cKO mice. **(E)** Representative pharmacological profile of oxygen consumption rate (OCR) in mitochondria from the VN of Vglut2:Ndufs4cCT and Vglut2:Ndufs4cKO mice. Seahorse assay was run with the assay media containing 10 mM pyruvate and 5 mM malate. OCR was normalized to the total protein amount. **(F)** Respiratory capacities for mitochondria from the VN of Vglut2:Ndufs4cCT and Vglut2:Ndufs4cKO mice (calculated from Figure 3E). Data are presented as the mean ± SEM. Statistical analysis was performed using an unpaired *t*-test (*P < 0.05, **P < 0.01, ****P < 0.0001). **(G, H)** OCR of VN mitochondria was measured in the presence of pyruvate/malate, succinate/rotenone, palmitoyl-carnitine/malate, following the addition of ADP, oligomycin, FCCP, and antimycin. Representative OCR results obtained with the addition of ADP **(G)** and FCCP **(H)** are shown. Data are presented as the mean ± SEM. Statistical analysis was performed using an unpaired *t*-test (*P < 0.05).

For global proteome analysis, a total of 34,297 spectra corresponding to 17,636 non-redundant peptides were identified through database search (1% FDR). Only peptides identified as unique (i.e., peptide sequences belonging to one single protein in the database) were considered. Overall, a total of 2,998 proteins were quantified from 15,102 non-redundant unique peptides. Among these, 22 proteins showed increased abundance (> 1.2-fold, P adj < 0.1), while 34 proteins presented reduced levels (< 0.8-fold, P adj < 0.1) in mitoTag preparations from Vglut2:Ndufs4cKO mice when compared to controls (Supplementary Table 1). Remarkably, most complex I subunits (36 out of 45 detected proteins) showed significantly reduced levels in NDUFS4-deficient mitochondria, except for the assembly factor NDUFAF2, which showed increased abundance in these mitochondrial preparations (Figures 3A,B). Constituents of the NADH dehydrogenase N-module (NDUFA2, NDUFA12, NDUFV1, NDUFV2, NDUFV3, NDUFS1, NDUFS6 and NDUFS4) were the most significantly decreased proteins in these mitochondria (Figure 3B). Constituents of the Q-module, which is located adjacent to N-module, and specific subunits of the P-module, were also reduced in Vglut2:Ndufs4cKO VN mitochondria (Figure 3B). Proteins with increased abundance in these mitochondria included the A, B and C-chain polypeptides of the complement subcomponent C1q (C1QA, C1QB, and C1QC), which are known to bind to the mitochondrial protein gC1qR (globular head C1q receptor or C1QBP) to modulate mitochondrial metabolism (Ling et al., 2018), and protein kinase C delta (PKCδ), a member of the protein kinase C family of serine- and threonine-specific protein kinases that translocates to mitochondria upon different stimuli (Majumder et al., 2001). Functional enrichment analysis for overrepresented gene ontology terms (Biological Process; Figure 3C) and pathways (Reactome; Figure 3D) confirmed complement-mediated signaling as the most enriched protein sets (terms) in the analysis of the proteins with increased abundance in mitochondria from the VN of Vglut2:Ndufs4cKO mice. Analysis of downregulated proteins in NDUFS4-deficient mitochondria resulted in highly significant categories confirming a role in mitochondrial function, and more specifically in respiratory electron transport, ATP synthesis and complex I biogenesis.

To explore the functional relevance of the proteomic alterations observed in Vglut2:Ndufs4cKO mice, we assessed mitochondrial respiration in mitochondria preparations from the VN. Mitochondrial oxygen consumption rate (OCR) was assessed by using a Seahorse XFp extracellular flux analyzer (Figure 3E). Mitochondria were first incubated with pyruvate and malate as respiratory complex I substrates (state II). Then, ADP was injected to induce phosphorylation (state III), followed by oligomycin to inhibit the ATP synthase (state IV) and the chemical uncoupler FCCP to induce maximal respiration (state IIIu). VN mitochondria from Vglut2:Ndufs4cKO mice exhibited a profound decrease in the OCR (state II, III and IIIu) compared to Vglut2:Ndufs4cCT mice. A more detailed analysis of VN mitochondria respiration (Figure 3F) revealed significantly reduced spare capacity, proton leak and ATP-linked respiration in Vglut2:Ndufs4cKO compared to control mice. Finally, whether the decreased VN mitochondria respiration was complex I mediated was investigated. To that end, we used saturating concentrations of specific substrates for complex I (pyruvate/malate), complex II (succinate/rotenone) and β-oxidation (palmitoyl-carnitine/malate) (Figures 3G,H). OCR results revealed that state III (ADP-induced) and state IIIu (FCCP-induced) respirations were significantly different between Vglut2:Ndufs4cKO and control mice for complex I substrates, and similar for complex II and β-oxidation substrates. Collectively, these data show a selective complex I-mediated dysfunction in mitochondria from the VN of Vglut2:Ndufs4cKO mice.

### Hyperacetylation of PDH and Complex V Subunits in VN Neurons of Vglut2:Ndufs4cKO Mice

**FIGURE 4:**
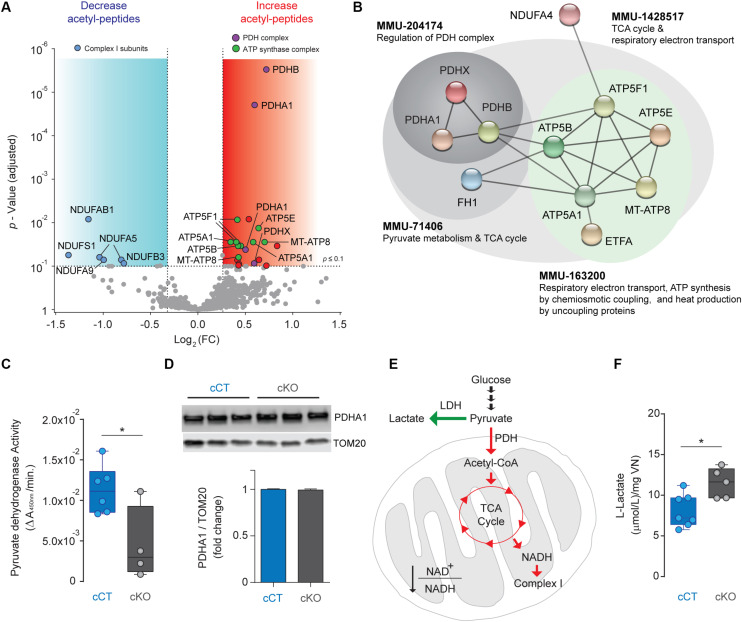
Analysis of the acetylome in NDUFS4-deficient mitochondria from the VN of Vglut2:Ndufs4cCT mice. **(A)** Volcano plot showing acetyl-peptides with decreased (< 0.8-fold, P adj < 0.1; blue area; *n* = 7) or increased abundance (> 1.2-fold, P adj < 0.1; red area; *n* = 20) in mitoTag preparations from the VN of Vglut2:Ndufs4cKO mice. **(B)** Protein-protein interaction network of proteins with differential increased acetylation in mitoTag preparations from the VN of Vglut2:Ndufs4cKO mice using STRING. **(C)** Pyruvate dehydrogenase activity in mitochondria isolated from the VN of Vglut2:Ndufs4cCT and Vglut2:Ndufs4cKO mice. Statistical analysis was performed using an unpaired *t*-test (*P < 0.05). **(D)** Western blot analysis for PDHA1 protein in mitochondria isolated from the VN of Vglut2:Ndufs4cCT and Vglut2:Ndufs4cKO mice. **(E)** Metabolic alterations in PDH activity signaling in the VN of Vglut2:Ndufs4cKO mice. Reduced PDH activity results in elevated levels of lactate. Green arrows show up-regulated pathways and red arrows denotes down-regulated pathways. LDH, lactate dehydrogenase; PDH, pyruvate dehydrogenase; TCA, tricarboxylic acid cycle. **(F)** L-Lactate concentrations in mitochondria isolated from the VN of Vglut2:Ndufs4cCT and Vglut2:Ndufs4cKO mice. Statistical analysis was performed using an unpaired *t*-test (*P < 0.05).

Mitochondrial complex I deficiency has been shown to lead to protein hyperacetylation in the heart (Karamanlidis et al., 2013). Hence, we next sought to assess changes in protein acetylation due to NDUFS4 deficiency in mitochondria from glutamatergic neurons in the VN. Acetylated proteins and their modification sites were identified after peptide labeling and affinity enrichment of lysine-acetylated (Kac) peptides by high-resolution LC-MSn in mitoTag preparations from Vglut2:Ndufs4cKO and Vglut2:Ndufs4cCT mice (Supplementary Table 2). A total of 3,445 spectra corresponding to 2,364 non-redundant peptides (928 proteins) were identified through database search (1% FDR). From these, 742 lysine acetylated peptides in 332 protein groups were accurately quantified. A total of 28 differentially acetylated peptides corresponding to 22 proteins were identified in mitoTag preparations from the VN of Vglut2:Ndufs4cKO mice when compared to controls (Figure 4A). Among these, 7 peptides showed decreased lysine acetylation (< 0.8-fold, P adj < 0.1), and most matched to complex I subunits including NDUFS1 (K709), NDUFAB1 (K88), NDUFA5 (K36 and K56), NDUFA9 (K189) and NDUFB3 (K40). On the other hand, 20 peptides showed increased lysine acetylation (> 1.2-fold, P adj < 0.1), 4 of them corresponding to subunits of the pyruvate dehydrogenase (PDH) complex (K321 on PDHA1, K354 on PDHB and K444 PDHX) and 9 corresponding to subunits of the ATP synthase complex (K48 and K54 on MT-ATP8, K239 and K539 on ATP5A1, K426 on ATP5B, K32 on ATP5E, and K162, K225, and K244 on ATP5F1). Functional protein association network analysis of proteins with increased acetylation using STRING (Figure 4B) showed that these fit into different Reactome pathway nodes, confirming regulation of PDH complex and respiratory electron transport and ATP synthesis. To correlate this increased acetylation in subunits of the PDH complex with changes in the activity of the enzyme, we next assessed mitochondrial preparations from Vglut2:Ndufs4cKO and control mice for PDH activity. Mitochondrial preparations from the VN of Vglut2:Ndufs4cKO mice showed reduced PDH activity when compared to controls (Figure 4C), which was not due to reduced PDHA1 levels, as assed by western blot analysis (Figure 4D). Reduced PDH activity may shunt pyruvate to lactate instead of entering mitochondria (Figure 4E), therefore, we next sought to define the concentration of lactate in the supernatants of mitochondria isolated from the VN of Vglut2:Ndufs4cCT and Vglut2:Ndufs4cKO mice (Figure 4F). Analysis showed significantly increased levels of lactate in samples from Vglut2:Ndufs4cKO mice, suggesting that glutamatergic neurons in Vglut2:Ndufs4cKO are more glycolytic than controls.

## Discussion

The mechanisms conferring neuronal vulnerability to LS are currently unknown. Mitochondrial dysfunction in unique cell populations leads to distinct clinical and pathological outcomes (Chen et al., 2017; Bolea et al., 2019). Furthermore, the type of dysfunction and the cellular metabolic status of the cell have been shown to initiate different stress-induced pathways (Mick et al., 2020). Therefore, cell type-specific analyses are needed to define the underlying cellular pathological mechanisms induced by mitochondrial dysfunction. Here, we have developed a tool that allows rapid, viral vector-based isolation of mitochondria with cell type specificity and in a spatiotemporally restricted fashion, with the overarching goal of identifying the underlying cellular mechanisms driving neuronal susceptibility to mitochondrial dysfunction. Recently, the urgent need to develop approaches to assess mitochondrial (dys)function in susceptible cell types has led to the generation of different genetic tools to isolate mitochondria with cell-type specificity (Bayraktar et al., 2019; Fecher et al., 2019). These are based on mouse lines that conditionally express, in a Cre recombinase-dependent manner, a tag (GFP or 3XHA-EGFP) that is targeted to the outer mitochondrial membrane to enable immunocapture of mitochondria from specific cell types. However, these approaches are limited by the possibility of unexpected transient expression of Cre recombinase during development in defined cell types (Song and Palmiter, 2018). A significant number of Cre-driver cell lines rely on temporally dynamic promoters that may be transiently active during development, leading to off-target recombination events that compromise the cell type specificity and significantly limiting the application of these tools (Lam et al., 2011; Sanz et al., 2015). Cre-dependent viral vector-based approaches such as our mitoTag assay combined with cell type-specific Cre driver mouse lines allow delivery of the transgene into the adult brain, therefore overcoming this important limitation. In addition, viral vector-based approaches avoid complex and time-consuming breeding strategies and enable temporal and regional control, therefore allowing rapid and anatomically restricted mitoTag assays.

Our proteomics data shows that NDUFS4 deficiency leads to mitochondria depleted of most of the subunits of the respiratory chain NADH dehydrogenase (complex I; CI). Complex I (CI; NADH: ubiquinone oxidoreductase; EC 1.6.5.3) is essential for oxidative energy metabolism as it contributes to the generation of the proton motive force needed for ATP synthesis (Hirst, 2013). This complex has a boot-shaped structure with a hydrophilic matrix arm and a hydrophobic membrane arm that is embedded in the inner mitochondrial membrane. These arms are subdivided into three structurally and functionally defined modules: the N-module (involved in NADH binding and oxidation; where NDUFS4 is located), the Q-module (for transfer of electrons along Fe-S clusters to ubiquinone), and the P-module (mediating proton pumping) (Formosa et al., 2018). Our results evidence that components of the N-module were the most significantly downregulated in mitochondria from the VN of Vglut2:Ndufs4cKO mice, indicating a critical role of this accessory subunit in the assembly and/or stability of the N-module component of CI, and confirming previous studies in LS patients harboring NDUFS4 mutations (Scacco et al., 2003) and *Ndufs4*-deficient mice (Calvaruso et al., 2012; Leong et al., 2012; Valsecchi et al., 2012). Furthermore, our data also shows an increase abundance of a specific assembly factor, NDUFAF2 (B17.2L), a molecular chaperone associated with a large subassembly of ∼830-kDa form of complex I and required in the late stage of CI assembly that is essential for the efficient assemblage of complex I (Ogilvie et al., 2005; Lazarou et al., 2007; Sanchez-Caballero et al., 2016). This increase may result from a compensatory attempt to keep complex I assembled upon NDUFS4 deficiency, and confirms recently published data (Adjobo-Hermans et al., 2020).

Our analysis also revealed mitochondrial association of characterized non-mitochondrial proteins upon NDUFS4 deficiency, which would remain unnoticed in a global analysis of the cellular proteome. Vglut2:Ndufs4cKO mitochondria showed increased abundance of the three different polypeptide chains that compose the complement subcomponent C1q. C1q has been shown to be present within the cell (Ten et al., 2010; Ghebrehiwet et al., 2012), and one of the receptors for C1q, the globular head C1q receptor (gC1qR), is a mitochondrial cell-surface protein known to modulate metabolism through association with C1q (Dedio et al., 1998; Kolev and Kemper, 2017). In this regard, an effect on spare respiratory capacity and oxygen consumption has been shown by gC1qR-bound C1q, which is known to enhance nuclear transcription of mitochondrial biogenesis genes (Ling et al., 2018). Association of C1q with NDUFS4-deficient mitochondria may be a mechanism of the cell to promote mitochondrial biogenesis to overcome mitochondrial dysfunction. Conversely, C1q has also been shown to mediate mitochondrial ROS production by cortical neurons in neonatal hypoxic-ischemic brain injury and that C1q-deficient brain mitochondria present preserved activity of the respiratory chain (Ten et al., 2010), suggesting tissue- and likely cell type-specific mitochondrial roles of complement factors. Therefore, the role of C1q association to NDUFS4-deficient mitochondria warrants further research.

Furthermore, our direct proteomics approach has detected increased abundance of PKCδ in mitoTag preparations from Vglut2:Ndufs4cKO mice, suggesting mitochondrial translocation of this kinase in NDUFS4-deficient vulnerable neuronal populations. In this regard, PKCδ has been shown to translocate to mitochondria upon different stressors and in different neurodegenerative pathologies, including oxidative (Majumder et al., 2001) and genotoxic stress (Lasfer et al., 2006), or cerebral ischemia (Dave et al., 2011). Targeting of PKCδ to mitochondria alters the mitochondrial membrane potential and induces apoptotic responses of cells to oxidative stress. Consistently, treatment with pan-PKC inhibitors increases survival and delays the onset of neurological symptoms in NDUFS4KO mice (Martin-Perez et al., 2019). Administration of isoform-specific PKCδ inhibitors to NDUFS4-deficient mice will define the relevance of this protein as a target for the treatment of mitochondrial disease.

CI-deficiency in the heart of conditional NDUFS4KO mice results in a NAD(H) redox imbalance, with a decreased NAD^+^/NADH ratio, inhibiting the activity of the mitochondrial sirtuin deacetylase SIRT3, and leading to protein hyperacetylation (Karamanlidis et al., 2013). However, the effect of NDUFS4-deficiency on the mitochondrial acetylome in neurons was unknown. Analysis of the mitochondrial acetylome in mitoTag preparations greatly reduces the complexity of the sample by avoiding the interference of heavily acetylated cytosolic and nuclear proteins. This results in increased identification of acetylated peptides corresponding to mitochondrial proteins. We report increased abundance of acetylated peptides corresponding to the pyruvate dehydrogenase (PDH) complex in NDUFS4-deficient mitochondria. PDH is involved in transforming pyruvate into acetyl-CoA that is subsequently used by both the citric acid cycle and oxidative phosphorylation to generate ATP. Increased acetylation at different subunits of the PDH complex anticipated reduced activity of the enzyme since acetylation is typically reported to inhibit the catalytic activity of mitochondrial enzymes (Ghanta et al., 2013). More specifically, it has been described that acetylation of PDHA1 at K321 reduce the activity of the enzyme. Noteworthy, K321 residue is a SIRT3 substrate (Fan et al., 2014; Wei et al., 2019), and SIRT3-mediated deacetylation of PDHA1 K321 increases PDH activity (Ozden et al., 2014), therefore evidencing that modifications in the acetylation status of this sole residue can direct the activity of the PDH complex. As suggested by our data, reduced PDH activity may be contributing to the Warburg effect by enhancing glycolysis over mitochondrial oxidation of pyruvate (Warburg, 1956; Kroemer and Pouyssegur, 2008; Vander Heiden et al., 2009). Hence, the reduction in PDH activity may be involved in the increased glycolysis observed in Ndufs4KO mice (Johnson et al., 2013) and contribute to the metabolic demise of *Ndufs4*-deficient cells. Noteworthy, mutations in the PDH gene are associated with X-linked Leigh syndrome (Ruhoy and Saneto, 2014), indicating its central role in mitochondrial function. Several ATP synthase complex subunits also showed increased acetylation in specific residues, including K239 and K539 on ATP5A1, and K426 on ATP5B. SIRT3 has also been shown to target these residues to regulate ATP synthase activity (Vassilopoulos et al., 2014), which will likely contribute to reduce ATP production in mitochondria from glutamatergic neurons in the VN of Vglut2:Ndufs4cKO mice (Wu et al., 2013).

To date, the marked heterogeneity in mitochondrial composition and function has limited our understanding of the underlying molecular determinants of the neuronal susceptibility to mitochondrial dysfunction. Therefore, it is paramount to study mitochondrial function in a genetically defined manner to unravel the pathogenic mechanisms of mitochondrial disease. Here, by combining mouse genetics and cell type-specific mitochondrial isolation, we have assessed the mitochondrial proteome of a neuronal population shown to play a key role in the pathophysiology of a well-established mouse model of mitochondrial disease. Our results underscore the marked consequences of CI-deficiency in the proteome of affected neurons, identifying the metabolic consequences of such alterations. Furthermore, we have identified a potential role for PDH, complex V, the complement factor C1q, and PKCδ in the pathology, paving the way for future studies of the therapeutic potential of modulating these pathways in the quest for effective treatments of mitochondrial disease.

## Data Availability Statement

The datasets generated for this study can be found in the ProteomeXchange Consortium (PRIDE) repository, http://proteomecentral.proteomexchange.org/cgi/GetDataset?ID =PXD016321.

## Ethics Statement

The animal study was reviewed and approved by Animal Care and Use Committee of the Universitat Autònoma de Barcelona (Barcelona, Spain).

## Author Contributions

AG, PP-D, MC, AU, MG-T, JA, ES, and AQ conducted the experiments and acquired and analyzed the data. AG, MC, JA, ES, and AQ designed the research studies. AG, ES, and AQ wrote the manuscript. AQ and JA provided reagents. ES and AQ coordinated the work. All authors contributed to the article and approved the submitted version.

## Conflict of Interest

The authors declare that the research was conducted in the absence of any commercial or financial relationships that could be construed as a potential conflict of interest.

## Footnotes

1 http://proteomecentral.proteomexchange.org

2 http://webgestalt.org/

3 https://string-db.org/
